# Tongue Tuberculosis as a Complication of Pott’s Disease in a Patient on Systemic Steroid Therapy without Pulmonary Tuberculosis

**DOI:** 10.3390/medicina60081282

**Published:** 2024-08-08

**Authors:** Samuel Sevilla-Fuentes, Luis Ángel Mendoza-Vargas, José Francisco Araiza-Rodríguez, Bertha Berthaúd-González, Ramcés Falfán-Valencia, Brandon Bautista-Becerril

**Affiliations:** 1 Hospital General de Zona 1 “Emilio Varela Luján”, Zacatecas 98000, Mexico; 2 Hospital General “Dr. Manuel Gea González”, Mexico City 14080, Mexico; 3 Hospital General de Zacatecas “Luz González Cosío”, Zacatecas 98160, Mexico; 4 Laboratorio HLA, Instituto Nacional de Enfermedades Respiratorias Ismael Cosío Villegas, Mexico City 14080, Mexico; dcb_rfalfanv@hotmail.com; 5 Sección de Estudios de Posgrado e Investigación, Escuela Superior de Medicina, Instituto Politécnico Nacional, Mexico City 11340, Mexico

**Keywords:** tuberculosis, tongue tuberculosis, Pott’s disease, mycobacterium infections, case report

## Abstract

A 78-year-old man with a previous diagnosis of rheumatoid arthritis on prolonged treatment with corticosteroids presented with intense and progressive pain at the cervical level that prevented him from resting his head and walking, in addition to an ulcerative lesion covering 80% of the lingual area that was previously treated as oral candidiasis without improvement. On arrival, with no clinical or serological data of rheumatoid arthritis, immunosuppressive treatment was suspended, and a biopsy of the oral cavity was requested, confirming the diagnosis of lingual tuberculosis, an extremely rare disease, occurring in less than 1% of extrapulmonary cases. MRI of the cervical spine showed a crush fracture of the C6 and C7 bodies associated with spondylitis of probably infectious etiology that required surgical treatment, and histopathological studies confirmed Pott’s disease. The patient displayed no evidence of pulmonary tuberculosis from arrival until the end of the follow-up.

## 1. Introduction

Tuberculosis (TB), caused by the Mycobacterium tuberculosis complex, is one of the oldest known diseases affecting humans and a leading cause of death worldwide [1]. Although substantial reductions in the number of people seeking and receiving TB diagnosis, treatment, and care were reported during the COVID-19 pandemic due to the overall response to COVID-19 [2], in 2021, there was a notable increase in the number of reported cases of tuberculosis (TB). By 2024, approximately 3 million individuals with active TB were undiagnosed and unreported to public health authorities, representing a significant proportion of TB-related deaths. Moreover, they continue to contribute to the ongoing pandemic of this disease by acting as a reservoir for the pathogen [3]. Tuberculosis primarily affects the lungs but can also affect other organs and tissues of the body, known as extrapulmonary TB (EPTB). EPTB accounts for 15% of newly diagnosed TB cases worldwide. The most affected organs are the kidneys, lymph nodes, liver, and bones. The oral cavity is an extremely rare site of involvement, with an incidence of less than 14% worldwide, and lingual presentations are even rarer, occurring in less than 1% of cases [4,5,6]. The diagnosis of lingual tuberculosis is challenging because the lesions are difficult to differentiate from other ulcerative conditions of the oral mucosa, such as oral candidiasis, proliferative verrucous leukoplakia, sarcoidosis, Wegener’s granulomatosis, Bechet’s disease, lichen planus, or lupus [7]. Lingual tuberculosis as a complication of Pott’s disease without pulmonary involvement is an exceedingly uncommon occurrence, to the extent that there is no precedent for a similar case. Consequently, the present case is particularly noteworthy and illustrates the importance of a multidisciplinary approach and appropriate treatment to prevent complications and minimize the spread of infection in the community.

## 2. Case Presentation

**Figure 1:**
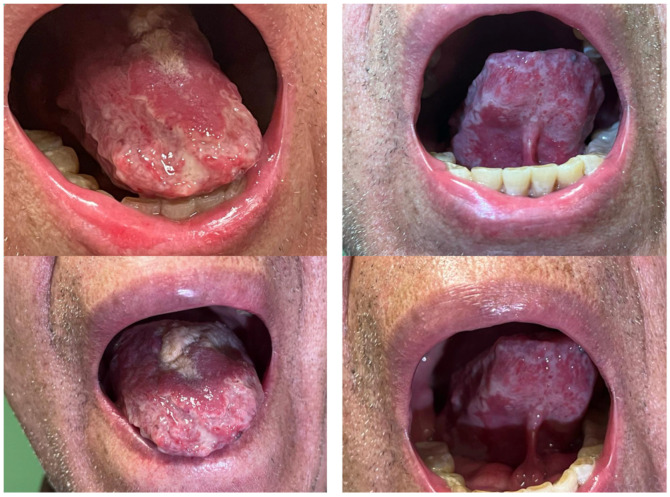
Ulcerative tongue lesions secondary to TB.

The patient is a 78-year-old male, currently retired, with a history of systemic arterial hypertension for 5 years and on treatment with Bisoprolol, with no history of chronic pulmonary diseases or smoking; the patient has received a complete vaccination regimen, including BCG, and had no recent contact with individuals diagnosed with tuberculosis or exhibiting signs and symptoms consistent with the disease; moreover, the patient had not undergone any prior immunodiagnostic testing for TB. The patient had joint pain, diagnosed as rheumatoid arthritis, for more than 6 months, and was on treatment with immunosuppressive drugs (Leflunomide and Prednisone) for 4 months. Less than 1 month ago, the patient also noticed the appearance of an ulcerative lesion with clean, white edges approximately 2 cm in diameter on the dorsum of the tongue, which caused him pain and made it difficult for him to eat; this lesion was treated as candidiasis and viral stomatitis. The patient exhibited no improvement in joint pain with treatment and continued with a weight loss of more than 10% of body mass in less than a month, as well as intense and progressive pain at the cervical level at C4–C6 and inability to support the head, which made walking difficult. The lingual ulcer continued to grow and spread rapidly to both sides of the tongue, causing intense pain and making it impossible to swallow (Figure 1), which is why he came to our department.

On arrival, the patient’s vital signs were within normal ranges; laboratory studies showed elevated acute phase reactants and marked neutrophilia. All other results were unchanged (Supplementary Table S1); clinically, he had no data suggestive of inflammatory arthropathy (absence of acute synovitis, morning stiffness >30 min, negative rheumatoid factor (RF) 12 IU/mL and anti-cyclic citrullinated peptide antibodies (Anti-CCP) 0.87 U/mL); but there was evidence of primary osteoarthrosis in the presence of Bouchard and Heberden nodules in the proximal and distal interphalangeal joints, respectively, morning stiffness <30 min, cramps in flexion and reduced range of motion in some joints, so the diagnosis of rheumatoid arthritis was ruled out and steroid treatment was discontinued. No signs or symptoms of active pulmonary TB infection or radiographic evidence of active or latent pulmonary infection were detected during the diagnostic and follow-up process (Supplementary Figure S1).

**Figure 2:**
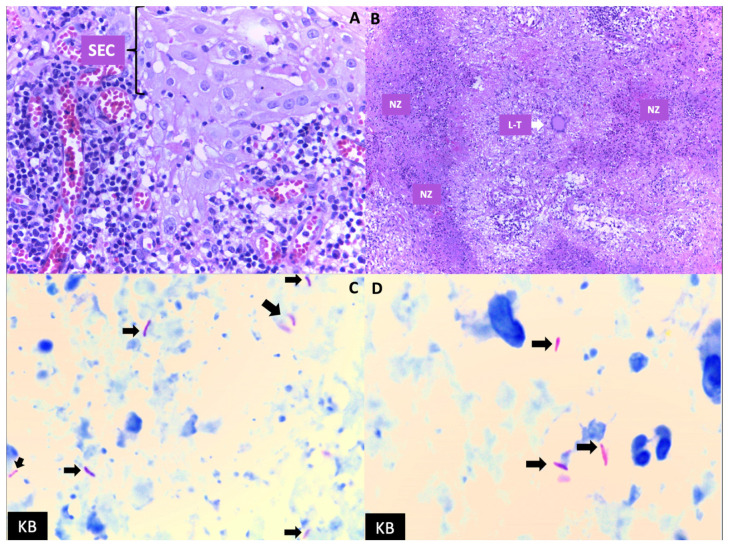
(**A**,**B**): Multinucleated Langerhans-type giant cells (L-G) and Necrosis Zones (NZs) on hematoxylin–eosin staining. (**C**,**D**): Caseating granulomas and Koch Bacilli (KB) on Ziehl–Neelsen stain. SEC: Squamous Epithelium of Oral Cavity. Black Arrows: Koch Bacilli.

Due to the persistence of the lesion in the oral cavity, a multidisciplinary approach was initiated, and the patient was presented to the surgery department for a lingual incisional biopsy, which the surgical pathology department would later interpret and use to establish an anatomopathological diagnosis. The biopsy showed the presence of multinucleated Langerhans-type giant cells on hematoxylin–eosin (H&E) staining (Figure 2A,B), and caseating granulomas and acid-fast bacilli on Ziehl–Neelsen (ZN) staining (Figure 2C,D), suggestive of Mycobacterium infection.

**Figure 3:**
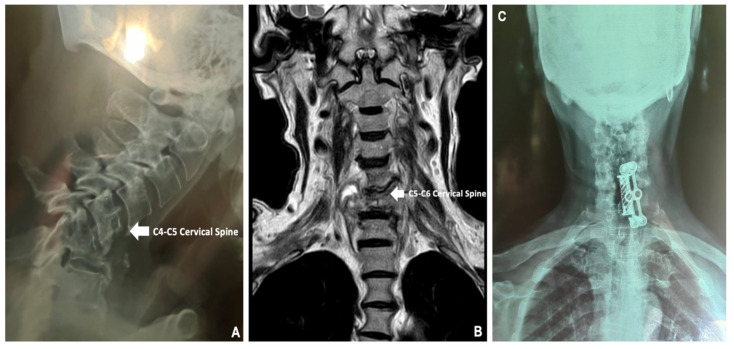
(**A**): Lateral X-ray of the cervical spine showing crushing of the C5 and C6 vertebral bodies. (**B**): Simple MRI up to T6, showing crushing of the C6 and C7 cervical bodies. (**C**): Anterior cervical spine fixation with threaded basket and automatic titanium locking.

Following these findings, a GeneXpert MTB/RIF expectoration molecular biology test was performed and confirmed the presence of Mycobacterium tuberculosis complex without rifampicin resistance in the *rpoβ* gene, so treatment with pyrazinamide, ethambutol, rifampicin and isoniazid was started immediately. For evaluation of the generalized pain in the cervical area, which compromised mobility and caused gait disturbances, inflammatory biomarkers were requested, which revealed a C-reactive protein (CRP) concentration of 68.39 mg/L, Erythrocyte Sedimentation Rate (ESR) of 50 mm/H, and Uric Acid (UA) concentration of 12.2 mg/dL; in addition, an anteroposterior and lateral X-ray of the cervical spine was performed, where there was evidence of crushing of the vertebral body of C5 and C6 (Figure 3A), which was complemented with an MRI of the cervical spine in simple phase to T6, where a crush fracture of the C6 and C7 bodies was reported, with changes in the intensity of the intersomatic disc, in addition to an anterior paravertebral and retropharyngeal component, conditioned by narrow cervical canal C5–C6 and C6–C7, with bilateral C6 and C7 radiculopathy, associated with spondylitis (Figure 3B). Images were suggestive of granulomatous infectious etiology, for which treatment measures were reinforced with oral pyridoxine 50 mg daily, pregabalin 150 mg daily and etoricoxib 90 mg daily, with no adverse events observed during the treatment administration. Finally, an assessment was requested by surgery, in which it was decided to place an anterior fixation of the cervical spine with a threaded basket and an automatic titanium block to improve the patient’s prognosis (Figure 3C). During surgery, a bone sample was obtained from the C6 and C7 bodies, which was examined by histopathology, where the final diagnosis of Pott’s disease was made.

Importantly, as soon as the diagnosis of rheumatoid arthritis was ruled out, corticosteroid treatment was also immediately discontinued to eliminate the risk of severe complications due to immunocompromise secondary to systemic corticosteroids.

**Figure 4:**
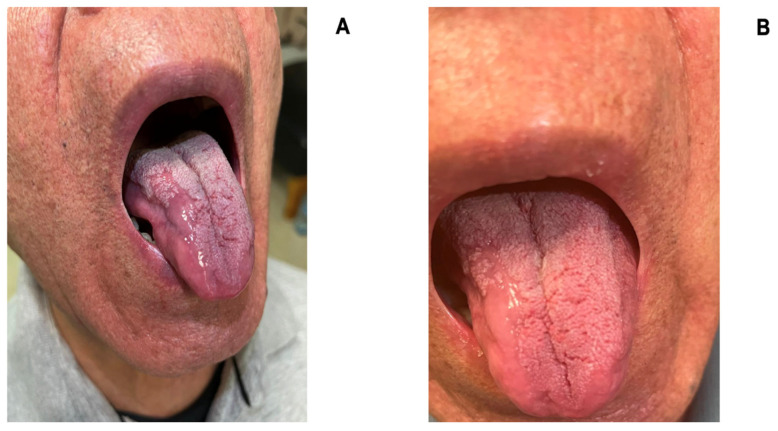
Ulcerative tongue lesion 6 months (**A**) and 12 months (**B**) after the pharmacological treatment.

Regarding the ulcerative lesion of the tongue, 6 months after the initiation of treatment with oral therapy, a significant improvement was observed, with a decrease in the size of the ulcerative lesions and pain control allowing swallowing (Figure 4A), and 12 months after treatment, we found an improvement in more than 90% of the ulcers and no pain on swallowing (Figure 4B). At 12 months after cervical fixation, the patient is ambulatory with adequate pain control and improved cervical mobility. He is able to hold his head at will, with adequate cervical and upper limb sensation, preserved range of motion, and slightly decreased upper limb strength (Daniels Scale 4) after surgery.

## 3. Discussion

Nearly two billion people (about a quarter of the world’s population) are estimated to be infected with Mycobacterium tuberculosis. In 2018, approximately 10 million people became ill with TB, and 1.5 million died; however, following the COVID-19 pandemic worldwide, there was an increase in reported deaths from tuberculosis in 2021 [2,7]. The rapid diagnosis of active TB facilitates timely therapeutic intervention and minimizes community transmission [8]. Oral lesions caused by TB are extremely rare, occurring in less than 1% of extrapulmonary cases [5]. To our knowledge, this is the first case where the initial TB infection was Pott’s disease, which spread to the oral cavity, causing lingual TB in the patient and later to the pulmonary tract, which makes the presentation of this case much rarer.

The first written record of oral tuberculosis was in 1888 about a man with pulmonary tuberculosis disease and ulcerative lesions that, until that moment, were not known how to categorize [9]. Since that time, a limited number of documented cases of lingual TB have been reported. A systematic review conducted by de Farias and colleagues in 2023 revealed that, between 1907 and 2020, approximately 300 cases of oral TB have been reported, with only 80 cases occurring in the lingual location [10]. Moreover, a systematic review conducted by Kakisi and colleagues identified 145 cases of oral tuberculosis between 1950 and 2009, of which only 47 occurred at a lingual site [11]. To the best of our knowledge, this is the first instance in which both lingual TB and Pott’s disease without active pulmonary infection have been described simultaneously.

The patient presented in this clinical case had multiple risk factors for the development of TB, which are consistent with those described in the most current literature. One of them is the immune compromise by prolonged or intense use of steroids. The patient used these drugs for around 75 days, and it has been demonstrated that every 1 mg of prednisolone equivalent dose per day increases the risk of immune compromise by between 1.09 and 1.12 times [12]. Likewise, an accumulated dose of 1000 mg (equivalent dose of prednisone) confers an 8 times higher risk of acquiring tuberculosis [13]. The patient currently resides in an urban environment, and it has been described that this could increase the risk of TB by up to 2.5 times [14]. In addition to the risk factors mentioned above and the patient’s lack of recognition of having been in contact with individuals with tuberculosis, we hypothesize that the patient was a carrier of a latent infection from an unidentified source. Although the precise cause remains uncertain, the patient’s immunosuppression probably led to the reactivation of the latent TB infection, which subsequently disseminated through the hematological and lymphatic systems, ultimately affecting multiple organs, including the bones and lingual mucous membranes. It is important to remember that our patient had been diagnosed with and treated for candidiasis and viral stomatitis without improvement, so it is essential to mention that despite its rarity, lingual/oral TB should be considered in the differential diagnosis of these cases since tuberculous lesions in the oral cavity may be the only manifestation of primary or secondary disease. In the following table, we offer a series of differential diagnoses to be considered (Table 1).

Finally, we emphasize the important learnings that long-term systemic steroid use and immunosuppressive therapies should always be very well justified and controlled while the patient is using them, as using them when not necessary increases the risk of life-threatening disease; furthermore, the multidisciplinary work of health personnel is vital for an early and directed diagnostic and therapeutic approach to reduce the probability of errors and increase the probability of success in the patient.

## 4. Conclusions

Tuberculosis is still an important disease in our environment, and we should always be on the lookout for it. Although lingual TB is of low incidence, the lack of response to antifungal treatment should always make us suspect alternative infectious diseases such as oral tuberculosis, as well as investigate the presence of HIV.

## Figures and Tables

**Table 1:** Differential diagnoses.

Differential diagnosis of lingual/oral TB
Moniliasis (thrush), neoplastic submucosal lesion, HIV complications, hairy leukoplakia, traumatic ulcer, aphthous ulcer, actinomycosis, syphilitic ulcer, Wegener’s granuloma, and oral carcinoma.
Differential diagnosis of Pott’s disease
Spondylitis of various etiologies (autoimmune, traumatic, antidegenerative), pathological fractures or metastatic cancer, multiple myeloma, spinal cord abscess, septic arthritis

## Data Availability

The data presented in this study are available on request from the corresponding author. The data are not publicly available due to privacy.

## Supplementary Materials

The following supporting information can be downloaded at: https://www.mdpi.com/article/10.3390/medicina60081282/s1, Figure S1: Chest x-ray of the patient on admission; Table S1: Clinical laboratories on patient arrival.
