# Supplementary material for: Tongue Tuberculosis as a Complication of Pott’s Disease in a Patient on Systemic Steroid Therapy without Pulmonary Tuberculosis
Source: Medicina (Kaunas). 2024 Aug 8;60(8):1282. doi: 10.3390/medicina60081282 (PMC11355980; doi:10.3390/medicina60081282)
Supplement: Supplementary file 1 [file medicina-60-01282-s001.zip › medicina-3121902-supplementary.pdf]

Supplementary material

Table S1: Clinical laboratories on patient arrival

**Blood Biometry and Blood Chemistry**
Hemoglobin 13 g/dL, BUN 7 mg/dL, Serum Creatinine 0.8 mg/dL, Glucose 82 g/dL, Cholesterol 195 mg/dL, Triglycerides 165 mg/dL, Uric Acid 12.2 mg/dL, Glucose: 88 mg/dL. Leukocytes 5200/mm^3, Neutrophils 4250/mm^3, Lymphocytes 800/mm^3, Eosinophils 20/mm^3, Monocytes 100/mm^3, Basophils 30/mm^3, Platelets 217 mcL,.
**Preliminary liver function tests**
Alanine aminotransferase (ALT) 19 U/L, Aspartate aminotransferase (AST) 21U/L, Globulins 2.5 g/dL, Alkaline Phosphatase 84 U/L.
**Rheumatoid profile**
C-Reactive Protein (CPR) 68.39 mg/L, Erythrocyte Sedimentation Rate (ESR) 50 mm/H, Rheumatoid Factor 12 IU/mL , Anti-CCP 0.87 U/mL.
**Extras**
General urine test with no abnormalities, HIV negative, HBV negative, HCV negative.

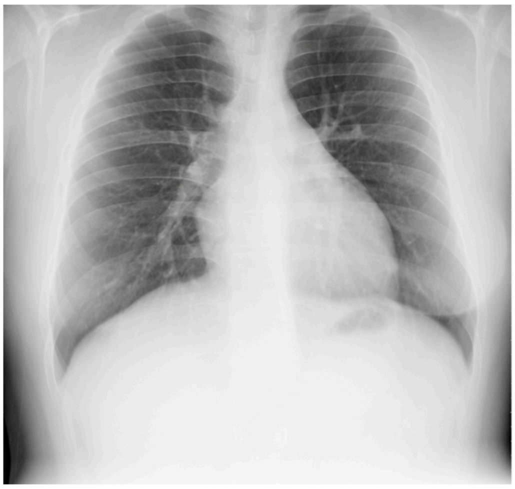

Figure S1: Chest x-ray of the patient on admission: lung parenchyma with air bronchogram of normal characteristics for age, no evidence of cavitation, micronodular disease or parahilar calcifications suggesting active or latent tuberculosis infection.
